# Coronavirus awareness and household hardship survey data for the CHAMPS HDSS network: Data collected between April 2021 and February 2022 in the Manhiça HDSS, Mozambique

**DOI:** 10.1016/j.dib.2024.110654

**Published:** 2024-06-21

**Authors:** Jonathan A. Muir, Teodimiro Matsena, Zachary J. Madewell, Fatima Keiri, Charfudin N. Sacoor, Edgar L. Jamisse, Elisio G. Xerinda, Aura M. Hunguana, Quique Bassat, Inacio Mandomando, Ariel Nhacolo, Solveig A. Cunningham

**Affiliations:** a Emory University, Atlanta, GA, United States; b Centro de Investigação em Saúde de Manhiça, Maputo, Mozambique; c Centers for Disease Control and Prevention, Atlanta, GA, United States; d ISGlobal - Hospital Clínic, Unversitat de Barcelona, Barcelona, Spain; e Institutó Catalana de Recerca I Estudis Avançats (ICREA), Barcelona, Spain; f Pediatrics Department, Hospital Sant Joan de Déu, Universitat de Barcelona, Barcelona, Spain; g CIBER de Epidemiología y Salud Pública, Instituto de Salud Carlos III, Spain; h Instituto Nacional de Saúde, Maputo, Mozambique

**Keywords:** Mozambique, Resilience, SARS-CoV-2, Southern Africa, Vulnerability

## Abstract

Data collection was implemented through an initiative by the Child Health and Mortality Prevention Surveillance (CHAMPS) Network to assess whether lockdowns and other social distancing policies during COVID-19 had implications for household economic status, maternal and child health, and healthcare accessibility for pregnant women and children. The data were collected from April 2021 until February 2022 from a population living in a rural community of Mozambique. This rural community is located within a Health and Demographic Surveillance System (HDSS) that operates in the Manhiça district of Maputo province. The survey instrument used for data collection was specifically designed to examine household awareness, knowledge, and prevalence of COVID-19; it was also designed to document hardships experienced by households during the pandemic period such as food insecurity, job losses and/or business closures of household members, and access to healthcare. The data are generalizable to a contiguous community in Manhiça, Mozambique of approximately 200,000 inhabitants.

Specifications Table

**Table:** 

Subject	Health Policy; Public Health
Specific subject area	Household awareness, knowledge and prevention of COVID-19; household level prevalence of COVID-19; and pandemic related household socioeconomic challenges and shocks
Type of data	Table
How the data were acquired	The survey instrument “Centro De Investigação em Saúde de Manhiça: Coronavirus Household Survey” was adapted from a standardized survey questionnaire “Harmonized COVID-19 Impact Questions for the CHAMPS HDSS Network” and used to acquire the data. The survey data, data collection instruments, and data dictionary are hosted on a *UNC Dataverse* data repository titled *CHAMPS Population Surveillance Dataverse* [1]. Fieldworkers followed a detailed data collection protocol that was approved by the Internal Scientific Committee (CCI) at the Manhiça Health Research Center (Centro de Investigação em Saúde de Manhiça - CISM) and Institutional Ethics Review Board for Health (CIBS), authorization reference number No Ref:CIBS-CISM/15/2021.
Data format	Raw
Description of data collection	Data collection took place within a predominantly rural, contiguous community in Manhiça, a district located in the Maputo Province of Mozambique that is about 80 km to the North of the capital city (also called Maputo) [[2], [3], [4], [5]]. In 1996, an HDSS was setup by the Manhiça Health Research Center, also known as the Centro de Investigação em Saúde de Manhiça (CISM). This HDSS currently spans 2380 km^2^, covers the entire Manhiça District, and follows a population of 201,845. Additional details about the Manhiça HDSS are published elsewhere [2,3]. Demographic and health-related data are collected from the population living within the HDSS during data collection rounds occurring one to two times per year. The demographic and health-related data collected during HDSS rounds are distinct from the data presented here, but are linkable therewith. All the households within the Manhiça HDSS were eligible to participate in the study. Study participants comprised either heads of households or their representatives—any household member 18 years old who had sufficient information about the household and all co-residing members and visitors.
Data source location	• Institution: Centro De Investigação em Saúde de Manhiça and Emory University • City/Town/Region: Manhiça / Maputo Province • Country: Mozambique
Data accessibility	Repository name: CHAMPS Population Surveillance Dataverse Data identification number: 10.15139/S3/SWK6BN Direct URL to data: https://dataverse.unc.edu/dataset.xhtml?persistentId=doi:10.15139/S3/SWK6BN Instructions for accessing these data: The data are accessible and available for download through the CHAMPS Population Surveillance Dataverse.

## 1 Value of the Data

• **Knowledge concerning COVID-19 Awareness**: These data provide a window into household awareness and familiarity with SARS-CoV-2 and COVID-19.
• **Understanding Socioeconomic Changes Concomitant with COVID-19**: The data offer information about the socioeconomic shocks experienced due to lockdowns and related policies implemented to mitigate disease spread during the pandemic period.
• **Implications for Child and Maternal Health**: These data elucidate potential repercussions on the health of mothers and children, highlighting challenges to accessing healthcare in a resource-limited setting.
• **Informed Policy Development**: The data can inform policy interventions, aiding public health researchers and policymakers in addressing the challenges stemming from the COVID-19 pandemic and associated lockdowns in resource-limited settings.
• **Versatile Analysis Possibilities**: Researchers can analyze these COVID-19-related data as a standalone dataset, or in combination with other data from the Manhiça HDSS, or as part of cross-site analyses with comparable data from other HDSS sites in the CHAMPS network [[6], [7], [8]]. These data could also be used in investigations that extend beyond the CHAMPS Network, after sufficient data harmonization, amplifying their utility and relevance across different research contexts.

## 2 Objective

The Coronavirus Awareness and Household Hardship Survey data were gathered in the Manhiça HDSS during a global health initiative among HDSS sites within CHAMPS [[8], [9], [10], [11], [12], [13], [14], [15]]. The goal was to examine household awareness and knowledge about the disease and to also examine potential implications and consequences of lockdowns and other policies aimed at mitigating the spread of COVID-19. More precisely, the data were collected to improve understanding of how these policies may have negatively affected households with potential impacts on livelihoods, food availability, and access to healthcare for children and pregnant women [4]. During data collection, respondents were prompted to consider their experiences since March 2020 to answer the survey questions. Data collection did not seek formally to obtain ascertainment of COVID-19 cases; COVID-19 lab tests were not included as part of this data collection process.

## 3 Data Description

Data (de-identified) from a survey of 33,087 households are publicly accessible via the data repository: *CHAMPS Population Surveillance Dataverse* in a downloadable file titled “COVID-19_Lockdown” that is accessible through the repository dataset: *COVID-19 Impact Data for the CHAMPS HDSS Network: Data from Manhiça, Mozambique*. Organization of the data within the file corresponds to the order of the modules from the survey instrument (as a temporal reference point to aid respondents in answering the survey questions, respondents were prompted to consider their experiences during the pandemic period, specifically since March of 2020):

▪ Identification of respondent
  - Example variables: respondent relationship to head of household
▪ Knowledge and practice regarding Coronavirus
  - Example variables: household awareness of and familiarity with strategies to prevent COVID-19 (e.g., hand washing, social distancing, and wearing a mask)
▪ History of symptoms and contact with people who had symptoms suggestive of Coronavirus
  - Example variables: ever-had symptoms of COVID-19 (colds, dry cough, cough with sputum, fevers, headache, sore throat, difficulty breathing, muscle aches, vomiting) and frequency of symptoms
▪ Impacts of COVID-19 on households
  - Example variables: household business closures, job loss of household members, and observations of increased prices for agricultural inputs, business inputs, and/or food
▪ Impacts on access to health care for household members
  - Example variables: routine check-ups; routine vaccination; routine antenatal care, delivery services, and postnatal care; malaria treatment; HIV treatment; clinical visits for illness; and accessing malnutrition services

Variable names in the data file follow the names listed in the data dictionary. Some survey questions allowed for respondents to select multiple answers; responses to these questions were separated into unique variables, each accompanied by descriptive variable labels for clarity.

The data dictionary is organized as two separate downloadable files:

▪ *Data Dictionary – Survey Information* provides detailed information about the survey instrument, organized into six columns (e.g., type, name, label, etc.); it describes the information collected through a survey version implemented electronically using Android-based devices.
▪ *Data Dictionary – Response Categories* provides the response categories for each question—responses are provided as they were originally programmed into the tablets in Portuguese, Mozambique's official language.

The survey questionnaire titled *Survey Instrument* is the original survey that was prepared for paper-based data gathering. The paper-based version of the instrument was later transferred into an electronic format and translated into Portuguese.

## 4 Experimental Design, Materials, and Methods

Data were gathered in the rural district of Manhiça in the Maputo province of Mozambique [[2], [3], [4]]. Demographic and health-related surveillance of the population has been ongoing since 1996 when an HDSS was established by CISM. The HDSS currently spans 2380 km^2^, covers the entire district of Manhiça, and maintains surveillance of a population of 201,845 [2]. The population is principally engaged in agriculture or as vendors, with a smaller segment employed in the district's two sugar factories, Maragra and Xinavane. The Manhiça health system comprises 19 health centers, a district hospital that is located in the village of Manhiça, and a rural hospital located in the administrative post of Xinavane [5].

Participants were recruited between April 2021 (before the wave of the Delta variant) and the peak of Omicron cases, in February 2022. Study participants comprised household heads or a qualifying representative (a household member aged 18 years or more with sufficient knowledge about the household and all co-residing members and visitors who entered the household since March 2020 until the date of the interview). All households within the Manhiça HDSS were eligible for participation in the study, which was implemented during routine HDSS round visits. The study adopted the definition of households and household members used by the HDSS. Households were defined as one or more individuals living together in the same dwelling or group of dwellings within Manhiça District and sharing domestic expenses, eating together, and recognizing one individual as their leader or representative. A household member was defined as a person who had resided in a dwelling within Manhiça District for three or more months or entered the study area with the intention of residence [2].

The survey instrument used for data collection followed state of the art survey design methods [16]. Steps in the design process included identifying central research concepts and questions, engaging in a brief literature review, and developing, adopting, and revising survey questions based on example questionnaires such as the one that the World Bank developed to assess economic and social consequences of the pandemic (i.e., the “High-Frequency Mobile Phone Surveys of Households to Assess the Impacts of COVID-19″ survey) [17,18]. A standardized survey instrument, originally prepared in English for implementation across sites within the CHAMPS network, was adapted to increase its suitability with local cultural and social contexts in Manhiça. For the Manhiça HDSS survey questionnaire, health authorities in the Manhiça district were actively engaged in adapting the questionnaire to local contexts and contributed additional questions to the instrument. The final questionnaire was organized into five sections: respondent identification, SARS-CoV-2 knowledge and practices; histories of symptoms and contacts with people that have symptoms suggestive of COVID-19; household impacts of COVID-19; and impacts on healthcare access for household members (see supplementary materials). The data collection instrument did not formally seek to obtain ascertainment of covid cases in the household, timing, and case definition; COVID-19 lab tests were not included as part of the data collection. The survey instrument prompted respondents to think about their experiences since March 2020 while answering questions related to hardships they may have experienced during the pandemic. Data collection was conducted electronically using tablets and a version of the instrument that was translated into Portuguese.

All communication with study participants was conducted using the respondent's preferred language. Similar to previous studies conducted by the CISM HDSS and social science team, if Portuguese was not a participant's preferred language, fieldworkers interpreted the questionnaires in situ. If a fieldworker was unable to communicate using the respondent's preferred language, an interpreter was identified within the household, or the household was revisited at another time by a fieldworker who was capable of communicating in the preferred language.

Cleaning and validation of the data (i.e., the process of identifying and correcting data errors throughout all stages of the data collection process) adhered to standard procedures for the HDSS [2,3,19]. Data collectors and supervisors received training about the study objectives, data confidentiality, and data gathering techniques. Data quality issues (e.g., as data inconsistencies, implausible values, or missing observations) were documented for reconciliation by data collectors through return visits. To evaluate data accuracy, a five percent sample of randomly selected households was re-visited to verify the information previously recorded. Data collection was authorized by the Internal Scientific Committee (CCI) at CISM and Institutional Ethics Review Board for Health (CIBS), reference number No Ref:CIBS-CISM/15/2021.

## Limitations

These data are specific to the households and individuals that were residing within the Manhiça HDSS at the time of data collection. Hence, these data are not generalizable outside of the geographic confines of the Manhiça HDSS. This limitation is common among studies that collected data from HDSS sites [14]. Given observational nature of the data, additional limitations include potential bias in the data such as recall bias and unmeasured variable bias. A cross-sectional study design was implemented for data collection. Lacking a longitudinal framing, the data did not capture temporal variations; it cannot inform analyses of potential causal relationships among indicators. This limits their utility for evaluating whether the severity and type of hardships associated with COVID-19 lockdowns changed over time [7]. This limitation could be addressed through creating longitudinal data that captured temporal variations; this could be accomplished through repeated administration of the survey questionnaire during future HDSS data collection rounds.

## Ethics Statements

This study followed the guidelines stipulated within the Declaration of Helsinki. Procedures that involved study participants, including the acquisition of informed consent, were approved by the Institutional Ethics Review Board for Health (CIBS); reference number No Ref:CIBS-CISM/15/2021. Respondents who were able to read and write provided written informed consent prior to participation. The informed consent statement was read and oral informed consent was obtained, documented, and witnessed from respondents who were unable to read or write. These procedures were approved by the Institutional Ethics Review Board for Health (CIBS), a review board affiliated to the National Bioethics Committee for Health (CNBS).

## CRediT Author Statement

Project administration provided by J.M., Z.M., A.N., C.S., Q.B., I.M, and S.C. Conceptualization performed by J.M., Z.M., A.N., C.S., and S.C. Methodology and investigation performed by J.M., Z.M., A.N., C.S., and S.C. Data curation carried out by A.N., C.S., E.J., E.X., T.M., and A.H. Validation performed by J.M., Z.M., F.K., A.N., C.S., and T.M. Writing original draft performed by J.M. Writing review & editing performed by all authors. Q.B., I.M, A.N., and S.C. provided supervision.

## Data Availability

COVID-19 Impact Data for the CHAMPS HDSS Network: Data from Manhiça, Mozambique (Original data) (Dataverse).
